# On the relation between the fields of Networked Music Performances, Ubiquitous Music, and Internet of Musical Things

**DOI:** 10.1007/s00779-022-01691-z

**Published:** 2022-10-05

**Authors:** Luca Turchet, Cristina Rottondi

**Affiliations:** 1 Department of Information Engineering and Computer Science, University of Trento, Via Sommarive 9, Trento, 38123 Italy; 2 Department of Electronics and Telecommunications, Politecnico di Torino, Corso Duca degli Abruzzi, 24, Turin, 10129 Italy

**Keywords:** Internet of Musical Things, Networked Music Performances, Ubiquitous Music

## Abstract

In the past two decades, we have witnessed the diffusion of an increasing number of technologies, products, and applications at the intersection of music and networking. As a result of the growing attention devoted by academy and industry to this area, three main research fields have emerged and progressively consolidated: the Networked Music Performances, Ubiquitous Music, and the Internet of Musical Things. Based on the review of the most relevant works in these fields, this paper attempts to delineate their differences and commonalities. The aim of this inquiry is helping avoid confusion between such fields and achieve a correct use of the terminology. A trend towards the convergence between such fields has already been identified, and it is plausible to expect that in the future their evolution will lead to a progressive blurring of the boundaries identified today.

## Introduction

In the past two decades and in particular in the last few years, we have witnessed the birth and diffusion of an increasing number of technologies, products, and applications at the intersection of music and networking [5, 10, 13, 21, 25, 35, 53]. As a result of the growing attention devoted by academy and industry to this area, three main research fields have emerged and progressively consolidated: the Networked Music Performances (NMP) [60], Ubiquitous Music (Ubimus) [42, 48], and lately the Internet of Musical Things (IoMusT) [76].

Today such fields represent established areas of research, which encompass both technical and artistic dimensions and involve different research communities, including Telecommunications, Sound and Music Computing, and Internet of Things. NMP, Ubimus, and IoMusT are also characterized by dedicated annual gatherings, such as the Ubiquitous Music Workshop (arrived this year to the 12th edition^1^) and the International Workshop on the Internet of Sounds (arrived this year to the 3rd edition^2^).

Whereas some of the topics faced by researchers and practitioners in these fields are radically different, others are common. This overlap sometimes may lead to confusion about the boundaries between such fields and about the areas in which they mostly operate, as well as to the inappropriate usage of the related terminology. A complicating factor is also that such research fields evolve with time. To address such confusion and miscommunication, in this paper, we identify and discuss commonalities and differences between these three research fields, as they are today. Our approach in attempting to disambiguate the concepts of NMP, IoMusT, and Ubimus is based on the analysis of current works in such three fields.

The rest of the paper is organized as follows: Section 2 surveys the related literature, whereas Section 3 provides an in-depth comparative analysis of NMP, IoMusT, and Ubimus. Finally, Section 4 sheds some light on potential future evolutions of the three research fields and provides conclusive remarks.

## Related work

### Networked Music Performances

Musicians have been fascinated by the idea of remote musical performances even before the birth of the Internet. As reported in [18], one of the first NMP experiments was performed by John Cage in the 1951, with the piece “Imaginary Landscape No. 4 for Twelve Radios.” The experiment used pairs of interconnected radio transistors as musical instruments [56], so that the two transistors could influence each other. This early trial, though heavily constrained by the technology of the time, can be considered as the first attempt to explore forms of networked musical practice. The rise of computers constituted a significant advancement towards the concrete possibility of more realistic musical interactions. One of the earliest networked music experiments with computers was performed in the late 1970s by a group named “The League of Automatic Music Composers” [55] and had the goal of influencing the performance of the group by exchanging messages between members using computers interconnected by a communication network.

**Table 1:** Feature comparison for some of the currently available HW/SW solutions for NMP

	ELK Aloha	Digital Stage	Jamulus	LOLA	JamKazam	SoundJack	JackTrip
Embedded systems support	$\checkmark$	$\varvec{X}$	$\varvec{X}$	$\checkmark$	($\checkmark$)	$\checkmark$	$\checkmark$
Uncompressed audio	$\checkmark$	($\checkmark$)	$\varvec{X}$	$\checkmark$	$\varvec{X}$	$\varvec{X}$	$\checkmark$
Video streaming support	$V$	$\checkmark$	$\varvec{X}$	$\checkmark$	$\varvec{X}$	$\checkmark$	$\varvec{X}$
Concert streaming to audience support	$\varvec{X}$	($\checkmark$)	$\varvec{X}$	($\checkmark$)	($\checkmark$)	($\checkmark$)	$\varvec{X}$
Supported by commodity ISP	$\checkmark$	$\checkmark$	$\checkmark$	($\checkmark$)	$\checkmark$	$\checkmark$	$\checkmark$

$\checkmark$ = supported; ($\checkmark$) = partially supported; $\varvec{X}$ = not supported

The 1990s played an important role in the evolution of NMP: in 1993, the University of Southern California Information Sciences Institute started experimenting with NMP over the Internet [63]. Four years later, in 1997, the group “The Hub” [14], which grew from the aforementioned group “The League of Automatic Music Composers,” experimented remote collaborations between the east and the west coasts of the USA sending MIDI data over the network. The choice to exchange message data instead of audio signals was forced by the limited channel bandwidth available at the time. A major step forward towards high-quality real-time remote musical interactions is represented by the development of high-speed and over-provisioned Internet backbones occurred in the past two decades. Within this time frame, a number of studies were devoted to the investigation of technical, perceptual, and artistic aspects of NMP. In the following, we report a brief overview of the most relevant ones. The interested reader may refer to [60] for a thorough survey.

#### Perceptual studies

NMP systems aim to achieve the same conditions as acoustic-instrumental on-site performances. The most fundamental issue in NMP applications is the latency introduced by the acquisition, packetization, and transmission of audio data through the network. A related issue is packet jitter (i.e., the latency variation between consecutive packets carrying audio data), which needs to be kept constant and as low as possible. Though some of such delay components (e.g., those that are hardware-dependent) are easily measurable or predictable, others are influenced by the physical distance between performers and by the overall traffic congestion conditions experienced by the network (e.g., propagation delays and queuing times at intermediate routers), which are time-variable and difficult to predict in realistic scenarios. To guarantee performative conditions as close as possible to those of traditional in-presence musical interactions, the mouth-to-ear delay perceived by musicians shall not exceed 20–30 ms, which correspond to the time taken by sound waves propagating in air to cover a distance of 8–10 m. Such distance is normally assumed to be the maximum tolerance threshold for the physical displacement among players in a room to ensure a stable interplay, in absence of further synchronization cues (e.g., as those provided by an orchestra conductor). Beyond such threshold, latency typically leads to a degradation of the performance quality, causing a tendency to tempo deceleration due to the fact that the counterpart is perceived to be “late.”

Several papers explored the effects of latency on the quality of remote musical performances, starting from hand-clapping experiments (see, e.g., [20, 27, 29]) and then taking in consideration other dimensions such as the timbral and spectral characteristics of the instruments being played, the rhythmic complexity of the executed piece, and the leader or follower role assumed by a player with respect to the others (as, e.g., in [7, 59, 63]). Typically, such experiments are conducted in a controlled environment where latency and packet jitter are artificially tuned by emulating specific network characteristics. Nevertheless, an increasing body of literature has investigated NMP in ecologically valid conditions [38]. Some NMP scenarios involving wireless communications have also been considered [33]. However, NMP applications leveraging wireless transmission are still heavily constrained by the technological limitations in terms of latency, since communication protocols must cope with much higher packet loss rates in comparison to cabled networks. 5G cellular networks promise to overcome such limitations in the near future, as they have already proved their effectiveness in supporting ultra low-latency applications [6, 57].

#### Technical issues

A second relevant issue in NMP is the recovery of audio artifacts due to lost or late packets carrying audio data through the telecommunication infrastructure. To minimize latency, the well-known retransmission mechanisms implemented at transport layer by the TCP protocol cannot be leveraged, as they can guarantee lossless and in-order delivery only at the price of introducing additional delays. Therefore, UDP must be leveraged as transport layer protocol, which ensures lightweight operations but does not provide any data integrity guarantee. Similarly, the usage of audio codecs capable of recovering transmission errors at the application layer is discouraged as the encoding/decoding process introduces further processing delay. It follows that, despite the huge existing literature corpus on error recovery techniques for audio data, ensuring professional audio quality for NMP applications in presence of packet losses without incurring in additional latency overheads is still an open research problem. Some audio codecs specifically tailored for ultralow-latency applications (such as OPUS [84]) have been developed, whereas, more recently, machine learning–based approaches for low-latency packet loss concealment have appeared [85].

A further source of audio artifacts is the drifting effect due to the imperfect synchronization of local clock oscillators, which may cause a deviation between the number of samples acquired by the sender and the number played by the receiver during a given time window, thus leading to buffer over/underruns. Though generally less impacting than packet loss and jitter, some studies have focused on compensating clock drifts by means of a tunable hardware oscillator circuit [90], whereas others propose the usage of a GPS-derived world clock [30].

Another promising research direction is to counteract the impact of mouth-to-ear latency by introducing an artificial metronome to provide audio cues to the musicians [8, 36], possibly integrating mechanisms to dynamically adapt to time-varying network conditions or to personalize the audio cues depending on the needs and preferences of the musicians, e.g., by introducing a virtual audio panning [37].

#### Artistic studies and demonstrations

Literature reports a considerable amount of publications dedicated to the assessment of musical practices over the network for both artistic and didactical purposes (see, e.g., [12, 22, 54]), which have particularly fluorished during the recent Sars-CoV-2 pandemic as a consequence of the social distancing countermeasures adopted to mitigate the virus spreading [32]. A series of telematic concerts of experimental electroacoustic improvisation named “Quarantine Concert Sessions” hosted by the Center for Computer Research of Music and Acoustics of Stanford University since March 2020 and involving musicians from three different continents constitutes one of the most recent examples [4].

#### HW/SW solutions for NMP

A number of either hardware or software-based solutions for NMP have been developed. Table 1 compares several currently available options, either at experimental or commercial stage. The interested reader can refer to [1–3, 16, 17, 28, 73] for additional details. Though the majority of them were originally conceived as software programs executable on general purpose machines and focused only on audio data streaming (video streaming was usually provided by running a videoconferencing application in parallel, with muted audio), recent advancements integrate video streaming and leverage dedicated hardware platforms that are specifically designed to minimize audio acquisition, processing, and buffering delays.

### Ubiquitous Music

Ubimus refers to music or musical activities that are supported by ubiquitous computing concepts and technology [62, 89], which embody the idea of all-pervasive and invisible computing present in our everyday life. The field is highly interdisciplinary and involves a wide range of approaches including artistic, technical, social, and environmental contexts. Ubimus can be placed at the intersection of music, computer science, education, and creativity studies [42, 48].

In [42], the following definition was proposed:

*Ubiquitous systems of human agents and material resources that afford musical activities through creativity support tools.*

The Ubimus field proposes to study how social interaction with mobile and distributed technologies can converge to form novel creativity support tools and musical practices [42]. Ubimus research and applications have pushed the boundaries of creative practice by involving non-professional musicians and even non-musicians as creative partners, and fostering the use of everyday settings for artistic and educational endeavors [40].

It is important to note that Ubimus is not statically linked to a particular set of applications and that it is an evolving area of research. While it has concerns that cross-cut into networking technologies and musical practice involving these, that in itself does not necessarily defines the area. The associated concept of Ubimus ecologies as explored in a recent publication [48] attempts to capture this, employing yet another metaphor imported from computing, where the word ecosystem has been employed to describe applications linked together in some way. Ubimus borrows this concept and extends it into a wider principle called *ecologies*: “the interrelated components of Ubimus, which may address musical, educational, technological, or creative concerns, or any intersection among these” [47]. Within these, we find, for instance, areas such as professional music and multimedia design, creation, and performance; sound and music computing technologies; the educational contexts; and issues of everyday creativity [24, 40, 41, 65].

### The Internet of Musical Things

The IoMusT is an emerging field that extends the Internet of Things paradigm to the musical domain [76]. The Internet of Things (IoT) relates to the network of “Things” [11]. These are computerized systems embedded in physical objects, which are connected to the Internet as well as are able to interact with each other and cooperate to reach common goals. Things are characterized by embedded electronics, wireless communication, sensing, and/or actuation capabilities. In the same way, the IoMusT refers to the network of “Musical Things,” which are computing devices embedded in physical objects dedicated to the production and/or reception of musical content.

A definition of IoMusT has been proposed in [76] considering the computer science perspective, as follows:

*the collection of ecosystems, networks, Musical Things, protocols and associated music-related information representations that enable services and applications related to musical content and activities, in physical and/or digital environments. Music-related information refers to data sensed and/or processed by a Musical Thing, and/or communicated to a human or another Musical Thing for musical purposes. A Musical Thing is a device capable of sensing, acquiring, actuating, exchanging, or processing data for musical purposes*

The IoMusT research field originates from the integration of many lines of existing research including ubimus [42], networked music performance systems [33, 60], Internet of Things [11], new interfaces for musical expression [39], music information retrieval [15], human-computer interaction [61], Musical XR [83], and participatory art [34].

Musical things, such as smart musical instruments or wearables, are connected by an infrastructure that enables multidirectional communication, both locally and remotely. The IoMusT technological infrastructure enables an ecosystem of interoperable devices that connect musicians with each other, as well as with audiences. This multiplies the interaction possibilities between a wide variety of stakeholders such as performers, composers, students, teachers, conductors, studio producers, live sound engineers, and audience members, both in co-located and remote settings [67, 91].

#### Musical things

Different kinds of Musical Things prototypes have been developed by the IoMusT community (see, e.g., [43, 92]), along with frameworks to connect them (see, e.g., [26, 31, 50, 87]).

One of the most prominent instances of Musical Things are the so-called smart musical instruments (SMIs). These are an emerging category of musical instruments characterized by sensors, actuators, wireless connectivity, and embedded intelligence [69]. Smart instruments are the result of the integration of various technologies including sensor- and actuator-based augmented instruments [52], IoT, embedded acoustic and electronic instruments [9], and NMP systems, as well as methods for sensor fusion, audio pattern recognition, and semantic audio. To date, only a few musical instruments that encompass the features of smart instruments exist in both industry and academy. Examples from industrial research are the Smart Guitar Lava Me 3 by Lava Music, the Smart Acoustic Guitar by HyVibe, and the Sensus Smart Guitar developed by Elk [75]. Examples in academic research are the Smart Cajón reported in [77] or the Smart Mandolin described in [68].

Together with the instruments, a number of innovative applications associated to them are also emerging. The system reported in [72] proposes a smart guitar system that uses the instrument as a hub for collaborative music making over a local wireless network. In such systems, performers using musical apps on smartphones produce sounds by wirelessly controlling the instrument’s sound engine, while the smart guitar player is actually playing and controlling other parts of the instrument’s sound engine. Another application for smart guitar has been developed to explore the use of distributed intelligence, via cloud computing and edge computing paradigms, for music learning and improvisation contexts [80]. Thanks to direct Internet connectivity and embedded processing, the instrument sends requests of wanted musical pieces to online music repositories and sonically reproduces the retrieved response for improvisation, composition, or learning purposes. Specifically, the search is performed using musical features, such as tempo and chords, which are extracted by the instrument capabilities, rather than utilizing the conventional text-based search criteria.

A radically different category of Musical Things is represented by wearables used for musical purposes. A relevant example in this space is represented by the so-called musical haptic wearables, a class of wearable devices embedding haptic stimulation, tracking of gestures and/or physiological parameters, and wireless connectivity features. On the one hand, such devices were conceived to enhance communication between performers as well as between performers and audience members by leveraging the sense of touch in both co-located and remote settings [71, 82]. On the other hand, they were devised to enrich musical experiences of audiences of music performances by integrating haptic stimulations, as well as provide new capabilities for creative participation thanks to embedded sensor interfaces [81].

Headsets for virtual or augmented reality can also be considered as Musical Things if used in networked musical applications and in conjunction with other Musical Things (see, e.g., [75]). However, this line of research has thus far received remarkably little attention [49, 83].

#### IoMusT ecosystems

An IoMusT ecosystem is composed of users involved in musical activities (e.g., musicians, audiences), as well as information and service providers. It forms around commonly used IoMusT hardware and software platforms as well as standards (e.g., the Elk Audio OS [73]). From the technological perspective, the core components of an IoMusT ecosystem are of three types: (1) Musical Things, (2) connectivity infrastructure (e.g., wireless sensor networks based on Wi-Fi [50, 74] or 5G [19]), (3) applications and services.

Recent endeavors in IoMusT research explored the creation of ecosystems around IoMusT technologies, proposing preliminary architectures based on Semantic Web technologies to foster interoperability across heterogeneous Musical Things. The semantically enriched IoMusT architecture reported in [78] relies on a semantic audio server, embedded audio systems, and edge computing techniques. In particular, the SPARQL Event Processing Architecture described in [58] was used as an interoperability enabler allowing multiple prototypes of Musical Things to cooperate. However, Semantic Web technologies are not suitable for IoMusT applications relying on real-time aspects, as the Semantic Web stack is oriented towards static scenarios, where information evolves at a low rate. To cope with this issue, Viola et al. improved the architecture reported in [78] by using CoAp, a lightweight IoT protocol for machine-to-machine communication [88]. Such architecture has been further improved and extended, leading to the Musical Semantic Event Processing Architecture (MUSEPA), a semantically based architecture designed to meet the IoMusT requirements of low-latency communication, discoverability, interoperability, and automatic inference [70]. MUSEPA uses at its core the Internet of Musical Things Ontology, an ontology dedicated to the representation of knowledge related to the IoMusT domain [79].

## Commonalities and differences

In this section, we counterpose features and exemplar systems in the three fields in order to identify commonalities and differences.

### NMP vs Ubimus

Surely Ubimus represents a much wider field than NMP. A comparison can be made across the following dimensions:

**Technological aspects.** NMP systems are just a technological enabler for certain Ubimus practices, such as mobile music in co-located settings. Nevertheless, to date, only a little body of Ubimus research has dealt with networked interactions, both between machines and between humans and machines. The extensive use of NMP systems in Ubimus research is yet to come.

**Temporal aspects.** NMP focuses on systems having a synchronous nature, i.e., systems allowing musicians to play together at a distance, in real time. Research on this field has traditionally focused on the development of techniques for reducing the impact on musicians of both network latency and its fluctuations, as well methods for increasing the audio quality due to packet losses. Conversely, Ubimus systems may be asynchronous.

**Spatial aspects.** Both Ubimus and NMP can focus on network-mediated interactions between stakeholders who are co-located or geographically displaced.

**Social aspects.** Most of the focus of Ubimus research is placed on the implications for stakeholders of ubiquitous music making (see, e.g., [23, 40]), focusing in particular on the concept of “Ubimus ecologies” [41, 48]. Comparatively, only a modest number of studies in NMP research has investigated social aspects (see, e.g., [64]).

### IoMusT vs NMP

As for the Ubimus field, NMP systems are also an essential component of the IoMusT. Nevertheless, a key distinguishing factor between the two fields is the concept of Musical Thing. Other aspects that distinguish the two fields are the following.

**Technological aspects.** To date, the majority of NMP applications are software programs that can run on commodity machines such as personal computers. Recently, dedicated hardware platforms for NMP have started appearing, which implement solutions specifically tailored for ultralow-latency audio acqusition and processing. Conversely, IoMusT necessarily requires dedicated hardware and may in turn leverage NMP applications to support the transmission of audio data through a networked infrastructure.

**Temporal aspects.** Though NMP may support several types of musical interactions, the majority of them require a synchronous interplay among the participants. Conversely, IoMusT encompasses a much more heterogeneous range of musical practices, for which the impact of the transmission delay of musical data is less disruptive. For some IoMusT applications, interactions can even be completely asynchronous.

**Spatial aspects.** Whereas NMP are inherently conceived to support musical interactions between subjects located in different geographical areas, where the networked streaming of audio data covers distances ranging from a few to thousands of kilometers (with some notable exceptions in the case of wireless networking [33]), IoMusT finds application also in much more restrained spatial dimensions, such as e.g. a single room.

**Social aspects.** Both NMP and IoMusT are conceived to support collaborative applications and services and thus natively foster social interactions among users. In particular, NMP can be exploited for remote teaching and didactical purposes. Several examples of virtual communities built around such services already exist. However, IoMusT allows for the acquisition, processing, and distribution of a much larger amount of data generated from heterogeneous devices, whereas NMP applications mainly focus on audio/video streams. This paves the way to future integration in the IoMusT ecosystem of big data frameworks for storage, processing, and management of the acquired information.

### Ubimus vs IoMusT

Historically, the IoMusT is a research area that has appeared after that of Ubimus. The IoMusT draws upon different strands of research, one of which is Ubimus. A comparison between the fields can be made across the following dimensions:

**Ubiquitous and non-ubiquitous activities.** The hardware and software platforms around which an IoMusT ecosystem is formed may support ubiquitous musical activities that take place outside of traditional venues such as concert halls, and that may involve the audience in the creative process. Nevertheless, in the IoMusT, both ubiquitous and non-ubiquitous musical activities are considered and may coexist. Indeed, the envisioned Musical Things as well as the IoMusT connectivity infrastructure have the potential to support also non-ubiquitous interactions (e.g., between musicians and audiences, such as those happening in conventional settings like concert halls) and a wider base of asynchronous interactions (e.g., between performers and producers, such as those happening in studios for music production).

**Stakeholders.** Whereas Ubimus focuses mainly on interactions involving performers, amateur musicians, and audience members, in the IoMusT paradigm, the interacting actors may also be many more. These include not only audiences and musicians (such as live sound engineers, conductors, composers, students, teachers, or studio producers) but also standardization bodies, musical institutions, publishers, studio recordings houses, and musical instruments manufacturers. Such heterogeneous stakeholders can co-exist and interact within IoMusT ecosystems. Furthermore, stakeholders in the IoMusT account also for musicians with impairments. For instance, IoMusT research has focused on accessible technologies for visually impaired performers [82]. Similar endeavors have not been the focus of Ubimus research thus far.

**Local and remote interactions.** To date, Ubimus systems and studies have mostly focused on co-located wireless interactions between stakeholders. Conversely, the IoMusT is more strongly oriented to remote interactions and to the development of systems that allow geographically dispersed musicians to play together (see, e.g., the NMP systems based on the HiFi Berry board developed by Elk and JackTrip Foundation [16, 73], or 5G architectures for IoMusT ecosystems [19]). Importantly, these interactions in the IoMusT may happen not only between stakeholders, but also between computer systems [50], where interoperability aspects play a crucial role [70, 78, 88]. With respect to this, Ubimus has not conducted research yet on the use of Semantic Web technologies, which are instead widely used also in the IoT field. Nevertheless, common to both fields is the fact that ubiquitous musical activities may or may not be networked. However, in the IoMusT vision, the emphasis is heavily put on networked musical interactions between human actors or between human actors and their machines.

**Professional audio equipment.** Thus far, Ubimus research has mostly devoted its attention to interactions supported by off-the-shelf devices like mobile phones, or do-it-yourself devices typical of the maker community [46, 66]. While some examples of use of professional audio equipment exist in Ubimus research (see, e.g., [93]), the IoMusT paradigm strongly relies on the use of professional audio equipment and advanced architectures (e.g., the Elk Audio OS operating system [73]).

**Multisensory aspects.** Another aspect that differentiates Ubimus from IoMusT one is the multisensory nature of the latter. While the visions of the Ubimus field proposed in [41, 42] and [48] mostly concern sonic content, in the IoMusT paradigm, the concept of musical content may encompass the use of Musical Things capable of providing their users with visual or haptic stimuli in addition to the sonic ones. Examples are musical haptic wearables for performers and audiences [71, 81], or networked virtual reality applications [49, 83], for instance for collaborative music creations [51].

**IoMusT ecosystems and Ubimus ecologies.** In recent years, Ubimus research has increasingly focused on the concept of ecologies [41, 48], i.e., relationship between stakeholders at various levels, which may or may not be mediated by the network. The IoMusT vision instead focuses on IoMusT ecosystems (see Section 2.3.2, and draws upon concepts and inquiries more typical of research on IoT ecosystems (e.g., at business, ethical, technological, and artistic levels) [76, 86].

## Discussion and conclusions

**Fig. 1:**
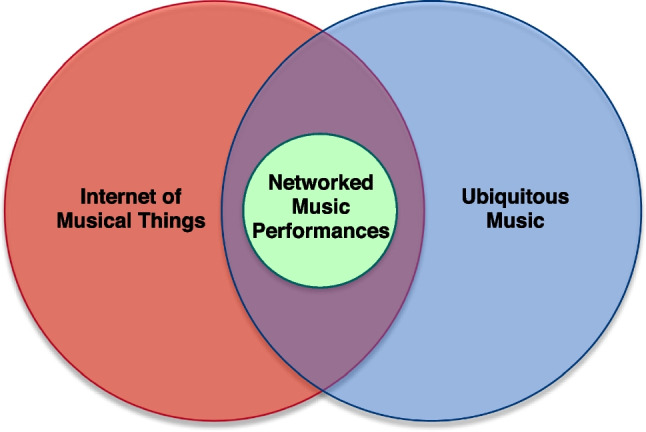
Relation between the fields of Internet of Musical Things, Ubiquitous Music, and Networked Music Performances

Figure 1 summarizes in a diagram the relationship between the three fields. As it is possible to notice, NMP is encompassed in both IoMusT and Ubimus, being a fundamental technological enabler for them. On the other hand, Ubimus and IoMusT are two independent fields of research that have many features in common, including part of their technological base.

Examples of studies and systems belonging to both Ubimus and IoMusT include those reported in [72, 80]. Examples of studies belonging to IoMusT but not to Ubimus are those reported in [19, 50, 70, 78, 79, 81]. Examples of studies belonging to Ubimus but not to IoMusT are [44, 45].

Despite such differences between IoMusT and Ubimus, it is possible to see a trend towards the convergence of these two fields. This is evident not only from the topics faced in the literature of the two fields, but also from the fact that the calls for papers of the International Workshop of the Internet of Sounds and that of Ubiquitous Music Workshop (which respectively are handled by the IoMusT and Ubimus communities) mention both the fields.

To date, the focus of IoMusT research has been mostly dedicated to engineering aspects, namely how to design and develop Musical Things as well as protocols and networking infrastructure for their interaction, whereas little attention has been devoted to social aspects or technological implications [80]. Conversely, a significant amount of Ubimus research has concentrated on contributions in terms of critical reflection of ubiquitous music making, especially considering creativity aspects. We believe that both fields would benefit from a wider integration in their focus of such complementarity of aspects, and it is plausible to expect that in the next few decades the IoMusT and Ubimus fields will progressively converge more than nowadays.

At the same time, Ubimus and even more IoMusT have the potential to bring benefits and opportunities to the NMP field. This is supported by the shift, witnessed in recent years, from desktop-based solutions for NMP (e.g., LOLA [28]) to dedicated embedded devices (e.g., Elk LIVE or JackTrip running on HiFi Berry [16, 73]). Other envisioned future directions concern the integration of motion sensors and haptic devices already adopted for IoMusT applications in NMP systems, e.g., to convey the gestural cues of a conductor or to complement remote teaching activities whenever direct visual feedback is not effective (e.g., in the case of blind players).

This paper attempted to delineate the differences and commonalities between the three fields of NMP, Ubimus, and IoMusT. Shedding light on these differences is useful to avoid confusing the three sectors and achieve a correct use of the terminology. However, we note that these fields are evolving and, therefore, some of the identified boundaries between them might become even more blurred in the future.

## Footnotes

1 https://easychair.org/cfp/ubimus2022

2 https://audiomostly.com/2022/call/cfiwis/
